# The production technology of mineral soda alumina glass: A perspective from microstructural analysis of glass beads in Iron Age Taiwan

**DOI:** 10.1371/journal.pone.0263986

**Published:** 2022-02-15

**Authors:** Kuan-Wen Wang, Yoshiyuki Iizuka, Caroline Jackson

**Affiliations:** 1 Institute of History and Philology, Academia Sinica, Nangang, Taipei, Taiwan; 2 Institute of Earth Sciences, Academia Sinica, Nangang, Taipei, Taiwan; 3 Department of Archaeology, University of Sheffield, Sheffield, United Kingdom; University at Buffalo - The State University of New York, UNITED STATES

## Abstract

Mineral soda alumina (m-Na-Al) glass is a common glass production group found around the Indo-Pacific region. In Iron Age Taiwan, its presence dates back to the early 1^st^ millennium AD. This research discusses m-Na-Al glass beads excavated from Iron Age sites in Taiwan. No production sites for m-Na-Al have been found, but microstructural analysis suggests m-Na-Al glass appears to originate around South Asia and is exchanged widely. SEM-EDS and EPMA were used to analyse red, orange, yellow, green and blue m-Na-Al glass. The microstructure of the glass shows the presence of plagioclase and alkali feldspar relics in the glass, suggesting a low manufacturing temperature. Copper-based colourants are identified in red, orange, blue and green glass, while lead tin oxide is used in yellow and green glass. It appears that various types of copper-containing raw materials were procured by craftspeople, and a self-reduction process for producing red and orange glass is tentatively proposed. Additionally, the microstructure of yellow glass reveals different colouring paths were used. These results increase our understanding of the selection of raw materials, and provide an impetus for further research on the cross craft interaction between glass and copper production.

## 1. Introduction

### 1.1. Mineral soda alumina glass sub-type 1: Elemental pattern and its distribution in the Indo-Pacific region in the 1^st^ millennium AD

Mineral soda alumina (m-Na-Al) glass is a silicate glass characterised by its high soda (~12–20 wt%) and high alumina (~5–15 wt%) contents. The low magnesia level (generally <1.5 wt%) suggests the soda flux was from mineral source. This high alumina glass is first reported by Brill [1], as a unique glass composition which suggests local glassmaking in South Asia. The sand used for producing m-Na-Al glass is possibly from river sand, with a composition similar to granite [2]. Unlike ancient glass from Late Bronze Age Egypt, Mesopotamia or Roman territories where relatively pure silica source was used, this high alumina glass was probably produced using a type of silicate sand which is less refined and contains impurities of alumina, iron oxide, lime, potash, enriched with titanium and trace levels of uranium [2]. The soda flux may be from *reh*, which is a natural soda efflorescence decomposed from mainly feldspar and plagioclase, in the form of carbonate, bicarbonate, sulphate and chloride, with small proportion of magnesium and calcium [3, Shahni (1950), cited in 4].

Five sub-types of m-Na-Al glass were identified by Dussubieux and colleagues [2], based principally on their magnesium (Mg), calcium (Ca), zirconium (Zr), barium (Ba), strontium (Sr) and uranium (U) contents, associated with their distribution over time and space. These sub-types are widespread across South Asia, Southeast Asia, East Asia, East Africa and Turkey, in periods from as early as 4^th^ century BC to 19^th^ century AD. Amongst the sub-groups, the artefact forms, colour distribution and glassworking methods also show differences. Two of these groups are of interest in glass found around the Indo-Pacific region in periods between mid-1^st^ millennium BC and 1^st^ millennium AD.

The two sub-types, m-Na-Al 1 and 3, primarily circulate around the Indian Ocean, the South China Sea and beyond. The sub-type 3 is an early chemical group dated to mid-1^st^ millennium BC. Its presence is restricted to the Thai-Malay peninsula around the South China Sea, with possible primary production in northeastern India [2,5,6]. The sub-type 1 is by far the largest sub-type. It is found in South Asia in 4^th^ century BC to 5^th^ century AD, and the production centre is thought to be near Sri Lanka [2]. Its counterpart is reported in Southeast Asia in the same period (although in small quantities in early periods) and circulation continues to 10^th^ century AD [7]. Within Taiwan, so far only m-Na-Al sub-type 1 has been identified from Iron Age sites [8,9], so this research focuses on the sub-type 1.

According to Dussubieux and colleagues, the m-Na-Al sub-type 1 has high barium (~931±432ppm), strontium (~373±145ppm), zirconium (~561±420ppm) but low uranium (~11±10ppm) (2). Beads and bracelets are the only artefact types seen in this group. Most of the glass beads are drawn beads and are found in opaque red, orange, yellow, green, black and translucent blue colours. White is rare, while dark blue is absent. Their physical appearance resembles the so-called Indo-Pacific monochrome drawn beads [7].

Similar distribution patterns of artefact types and colours is observed in Taiwan [9]. Beads here also comprise the majority of glass artefacts of a m-Na-Al 1 composition, in opaque red, orange, yellow, green or translucent blue colours, while bracelets are less common. Some black beads may be made of m-Na-Al glass sub-type 1, but have not been compositionally characterised. Within Taiwan, recent research further reveals a regional distribution of bead colours and styles at several Iron Age sites across the island, in particular the north and south areas [9]. It was suggested that the regional distribution illustrates the presence of inter- and intra-regional exchange systems at various scales and may be associated with certain economic and social practices in local communities [9].

In Southeast Asia, based on her research in the Mekong Delta, Carter [7,10] proposed that the wide spread of m-Na-Al glass sub-type 1 in early 1^st^ millennium indicates mass production of beads made of m-Na-Al glass. This broad circulation is associated with intensified interaction between Southeast Asia and South Asia, together with the presence of exchange networks and socio-political changes particularly in mainland Southeast Asian societies at multiple scales. The adoption of South Asian elements in cultural and socio-political contexts, however, is not evident in the archaeological record in prehistoric Taiwan. The circulation of m-Na-Al glass sub-type 1 in Taiwan may have been weighed more to the pre-existing South China Sea network built upon the Neolithic nephrite exchange [9,11]. The mechanism of glass exchange in economic contexts in Taiwan thus may take a different form from that in mainland Southeast Asia. This indicates that the expansion of the South China Sea network, as well as the interaction with the Bay of Bengal network to the west of Southeast Asia, in the Iron Age took place within complex, dynamic and multi-dimensional paths, for a long duration and at variable rates. More comprehensive discussion to the exchange mechanism of artefacts made of m-Na-Al sub-type 1 glass is found elsewhere [7,9,10,12].

### 1.2. Glass and bead manufacturing: Archaeological and ethnographic evidence

The manufacturing of glass can be divided into two stages: glassmaking and glassworking. Glassmaking involves the procurement of raw materials, the pre-treatment of raw materials (if any) and the process of transforming the raw materials (sand, flux and in some cases stabilisers) into raw glass. Glassworking contains the production of raw glass into glass artefacts, including vessels and ornaments. Glass colouring can take place during glassmaking or glassworking. The chemical compositions used for the identification of m-Na-Al glass sub-types are generally associated with the sand and flux, and therefore chemical analysis of this glass helps elucidate materials used and provenance of glassmaking.

Around the Indo-Pacific region, locating areas of primary production is mainly based on the identification of specific chemical groups (production groups) and their distribution patterns, although a few sites have yielded infrastructural evidence for glassmaking. The major compositional analysis has shown a broad tradition of using raw materials containing high alumina in glassmaking, while the minor and trace elements may indicate minute variations of raw materials procured from local areas. Present findings have suggested that the m-Na-Al sub-type 1 was made exclusively in South Asia. Archaeological evidence for primary production has been attested in Giribawa, Sri Lanka, where a glass furnace and raw glass showing an m-Na-Al sub-type 1 chemical composition has been reported [13,14], while in Southeast Asia, there are no archaeological finds to firmly suggest any stage of the production of m-Na-Al glass sub-type 1. This makes it a challenge to reconstruct the supply chain of this sub-type in the relatively long duration of the 1^st^ millennium.

Ethnographically, high alumina glassmaking has been documented in northern India, although not necessarily of a type parallel to the sub-type 1. Sode and Kock observed that high alumina glass was produced in 2 stages, firstly a low-temperature melted glass frit and secondly a fully melted raw glass. In the second stage, new batch (a processed mixture of sand and salt flux) can be poured into the molten frit and then melted together [15]. A low production temperature of around 900°C was observed for glassmaking here.

Most archaeological evidence across South and Southeast Asia, including Taiwan indicates secondary production only, particularly glass beadmaking. These include finds of semi-perforated beads, glass tube fragments and waste associated with different stages of glass beadmaking [7,16,17]. The bead styles are mostly the well-known Indo-Pacific beads. Our understanding on this type of beadmaking process is built up, in the first place, by Peter Francis’ archaeological and ethnographical observation [18,19], in which long glass tubes were drawn from a melted and cone-shaped glass lump inside the furnace. These long glass tubes were later cut into small pieces of beads. Over the past decades, Kanungo’s ethnoarchaeological studies at Papanaidupet have provided a more comprehensive picture for understanding the organisation of bead production [20]. The research shows that different stages of beadmaking may have been carried out in various locales, by different peoples, resulting in a dispersed distribution of beadmaking waste across wide areas near the workshop. This shows the complexity of identifying glass production workshops through archaeological evidence, as the presence of large lumps of bead finds may not necessarily point to glassmaking but glass beadmaking, and the latter may not be restricted in a single working space [20]. It is also noteworthy that the 20^th^ century ethnographic records from Papanaidupet suggest recycling or re-use of waste glass for glassmaking [20:19–31].

## 2. Objectives of this research

The wide distribution of m-Na-Al glass over time and space, together with the artefact types, colours and manufacturing methods, suggest a dynamic mode of the production and supply in various geographic regions through time. As yet, most discussion relating to the circulation and production of m-Na-Al glass is informed by the chemical composition relating to the characterisation of the geological sources of the primary raw materials. The lack of archaeological evidence for primary production, however, has made it hard to further discuss the organisation of production, despite ethnographic records providing parallels. More importantly, as part of the production process, the introduction of colouring agents, in what form they were introduced as raw materials and the relevant production technology have received limited attention, either within archaeological or ethnographic studies. The possibility of using various colouring agents in m-Na-Al glass production, or any technological practices or knowledge exchange of m-Na-Al glass colouring in specific regions or periods is seldom investigated.

The main purpose of this article is to demonstrate that, although there is no archaeological evidence of m-Na-Al glassmaking or glass colouring in Taiwan, a combination of chemical and microstructural analyses will yield more evidence related to production practices, in particular the use of different colouring agents. This enables us to infer the production technology of m-Na-Al glass around the Indo-Pacific region and to discuss the choice of raw materials and interactions between different craft technologies.

## 3. Research materials and methods

Most analyses of m-Na-Al glass beads utilise micro-destructive methods, principally LA-ICP-MS. The purpose of micro-destructive analysis is to reduce the damage to these tiny beads, but this can hinder the chance to examine any raw material relics or crystal phases in the microstructures of these opaque and strongly coloured glass beads. Here destructive sampling of the glass beads has been possible for microstructural analysis as many are fragments from multiple sites in Iron Age Taiwan.

**Fig 1:**
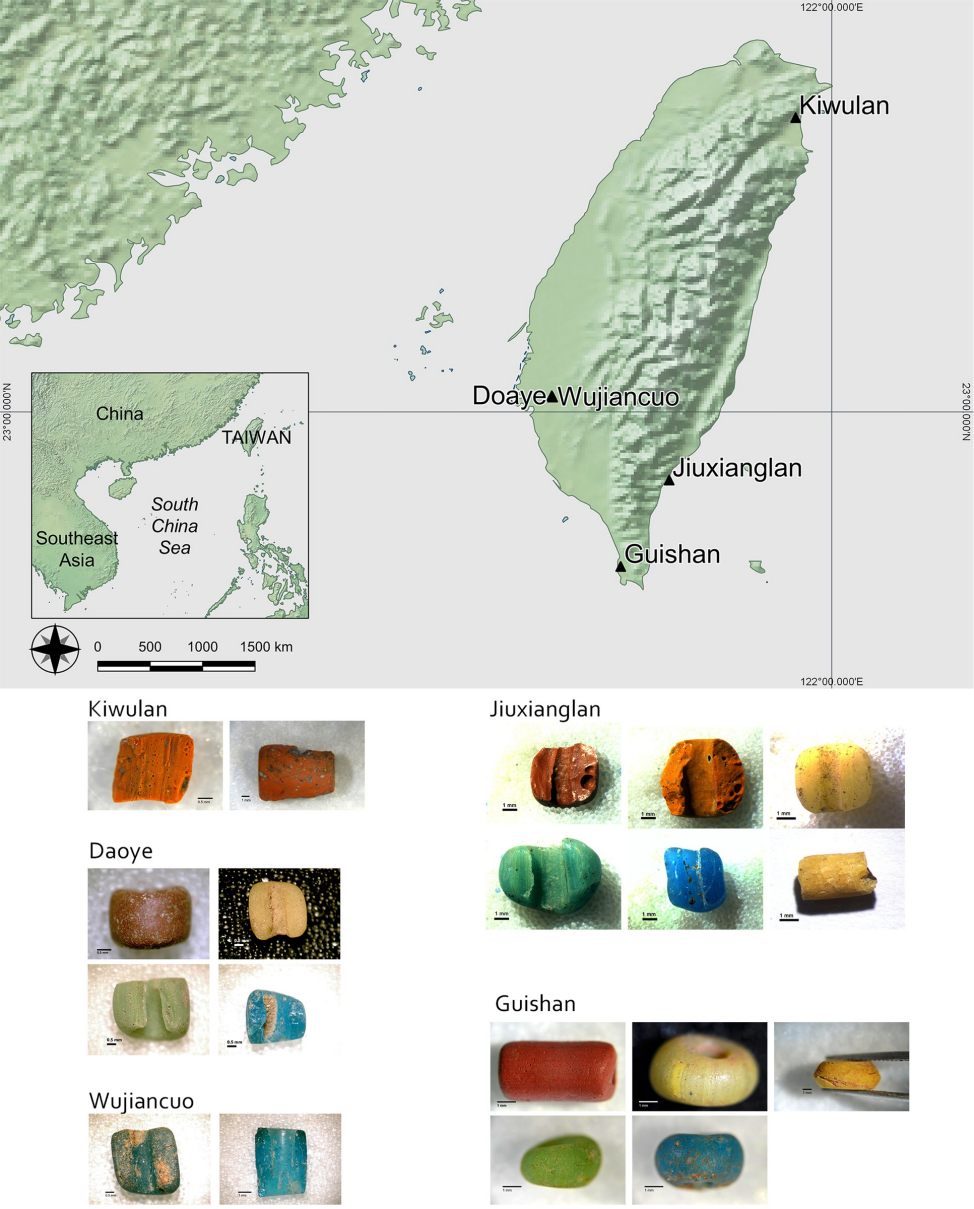
Map showing the location of study sites (above), and bead samples from each site (below). (Map made with Natural Earth. Free vector and raster map data @ naturalearthdata.com.).

A total of 63 glass bead samples from 5 Iron Age sites in Taiwan were analysed, including 5 from Kiwulan, 36 from Jiuxianglan, 8 from Daoye, 3 from Wujiancuo and 11 from Guishan (Table 1). This comprises a good range and combination of bead colours, covering red (n = 9), orange (n = 8), yellow (n = 15), green (n = 15) and blue (n = 16). Fig 1 presents the location of each site and photos of beads. Note that not all bead colours were found at all sites as shown in Table 1. Unfortunately a more precise chronology within a site is not possible to provide at this moment due to the lack of systematically dated stratified contexts.

**Table 1:** Summary of the study sites and the context of excavated glass beads.

Site	Chronology	Sample amount	Red	Orange	Yellow	Green	Blue
Kiwulan (Lower Cultural Layer)	7-12th C. AD	5		5 beads			
Jiuxianglan	2nd C. BC– 8th C. AD	36	6 beads	3 beads	9 beads 1 waste	9 beads	8 beads
Daoye	2-6th C. AD	8	1 bead		1 bead	3 beads	3 beads
Wujiancuo	5-8th C. AD	3					3 beads
Guishan	5-10th C. AD	11	2 beads		4 beads	3 beads	2 beads

The 5 sites show a wide chronology spanning over the 1^st^ millennium. The earliest is Jiuxianglan, from 2^nd^ century BC to 8^th^ century AD. Daoye falls in the 1^st^ half of 1^st^ millennium, Wujiancuo and Guishan is around mid-1^st^ millennium, and Kiwulan is roughly from the 2^nd^ half of 1^st^ millennium to early 2^nd^ millennium. Many sites overlap in dates thus provide a coherent and relatively uninterrupted chronological coverage. A detailed description for the archaeological context of each site can be found in Wang and Jackson [9].

Optical microscopy was used to observe any beadmaking marks left on bead surface. Microstructural and semi-quantitative chemical analysis was carried out by Scanning Electron Microscope (SEM: JEOL FE-SEM JSM-7100F) equipped with an Energy Dispersive Spectrometer (EDS: Oxford Instruments, Xmax-80), with an accelerating voltage of 15 kV, a probe current of 0.1 nA and a working distance of 10 mm. An electronprobe microanalyser (EPMA, JEOL JXA-8500F), equipped with wavelength dispersive X-ray spectrometers (WDS), was used for quantitative chemical analysis of the glass matrix. Fifteen elements (Si, Al, Na, K, Mg, Ca, Fe, Pb, Ba, Ti, Mn, Cu, Sn, Cl and S) were analysed, as oxides, with an accelerating voltage of 12 kV, a beam current of 6 nA and defocused beam diameter of 5 μm. A focused beam was used for crystal phases in glass matrix.

It should be noted that the performance of EDS detector is enough to identify elemental X-ray peaks with a lower beam energy setting (at 15 kV and 0.1 nA in this study). Whereas the analysis of glass composition by FE-EPMA used a 12 kV and 6 nA beam condition, which is a strong beam in comparison to EDS because it requires optimum X-ray intensities with reliable signal/noise (S/N) ratio for WDS counts. Furthermore, during quantitative analysis on glass samples, alkaline elemental migration, especially Na, may occur due to heating effect by the electron beam bombardment. To avoid the electron beam damage, we applied the same technique of analysis to study for volcanic glasses [e.g. 21].

Except for samples from Daoye and Wujuancuo, additional minor and trace elements were sought by Laser Ablation–Inductively Coupled Plasma—Mass Spectrometer (LA-ICP-MS), using an ICP-MS spectrometer (Agilent 7500a, USA) in conjunction with a New Wave UP213 laser ablation system, combined with a Nd:YAG laser at a wavelength of 213 nm.

The data quality is assessed by repeated analysis of Corning Glass Standards A, B, C, D and NIST 610, 612. Generally a good precision was seen, although the underestimation of MnO and Sb_2_O_5_ in Corning C are routinely observed. Full discussion on the analytical parameters, precision and accuracy can be found in [22].

## 4. Results

As only m-Na-Al glass sub-type 1 is identified in Taiwan, any following discussion referring to m-Na-Al glass refers to the sub-type 1.

### 4.1. Glass matrix (Matrix microstructure)

The full chemical compositional data can be found in supporting information (S1 Table). The base composition indicates that all samples are m-Na-Al glass, with Na_2_O from 11–22 wt%, Al_2_O_3_ between 6–15 wt% and SiO_2_ in 58–72 wt%. A low MgO (<1 wt%) content is observed in most samples, except for 2 Jiuxianglan samples and 1 Daoye sample which have MgO between 1.5–2 wt%. The minor and trace elemental patterns further confirm that all samples belong to the sub-type 1 defined by Dussubieux, Gratuze and Blet-Lemarquand [2], showing high Ba contents between 0.05–0.25 wt% and low U below 20 ppm.

The chemical composition suggests that a mineral soda flux was introduced with a sand containing aluminosilicate minerals for glassmaking. This can be supported by SEM-EDS microstructural analysis, as un-melted or partly melted feldspars are frequently found in the glass matrix. Alkali feldspar and plagioclase are relatively common, and show a solid solution enriched in soda, close to the albite endmember. Accessory minerals such as zircon is quite common, while quartz, ilmenite and other types of aluminosilicate minerals are occasionally found. It is therefore possible that the sand raw material already contained some soda (from feldspar) that can act as flux for glassmaking. However, the common presence of accessory minerals in the microstructure suggests that the sand may be poorly refined, and/or the components are variable. Pure sodium-rich feldspar contains around 65–70 wt% soda, under 20 wt% alumina and around 12 wt% soda. The glass composition here has silica contents close to 60 wt% and alumina around 10 wt%, suggesting greater proportion of pure silica than sodium-rich feldspar in the added sand. Therefore, an additional soda source may have to be introduced to facilitate melting.

**Fig 2:**
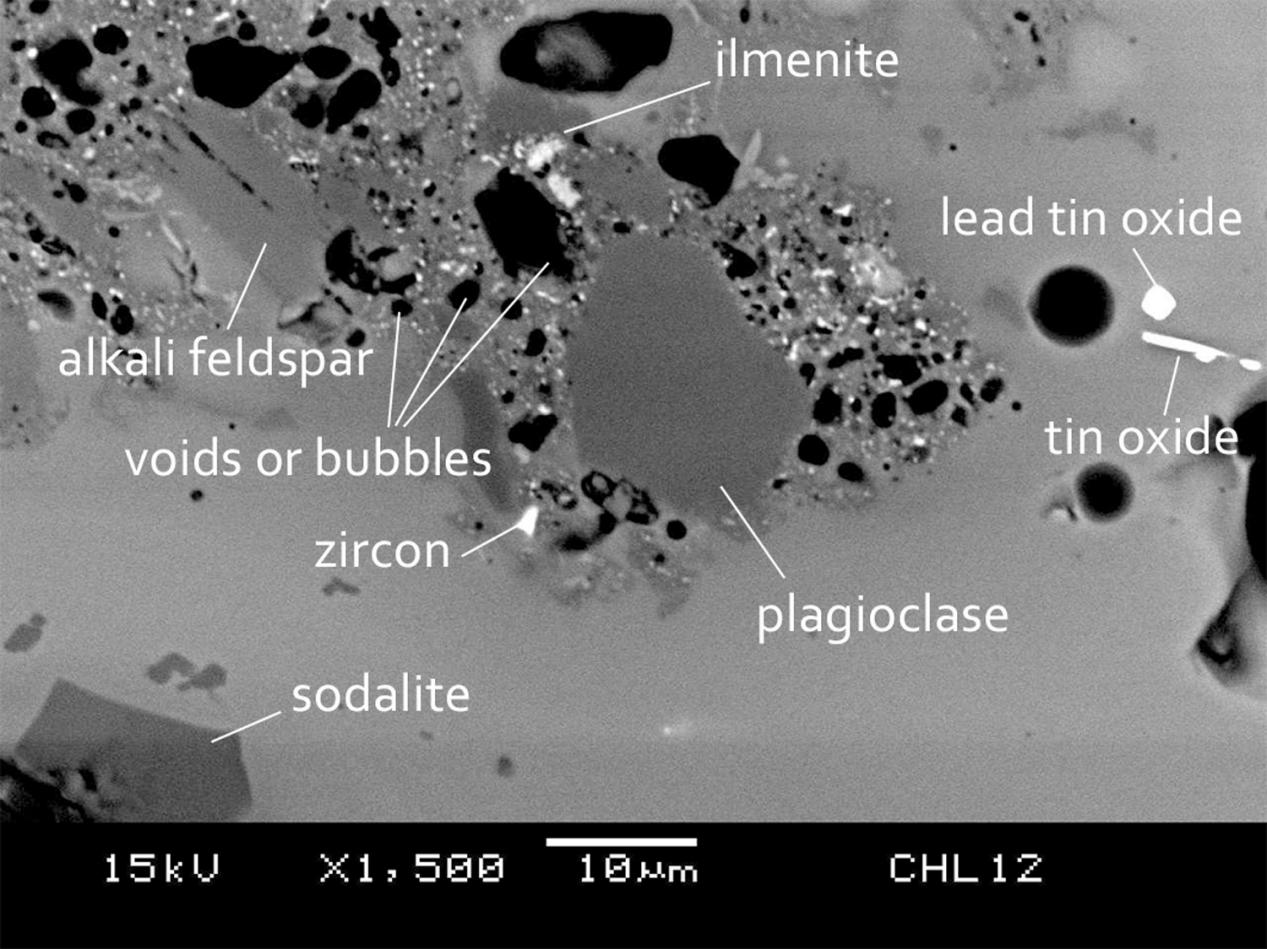
BSE image showing the heterogeneous microstructure of the glass matrix.

Fig 2 is a good example of the microstructure of m-Na-Al glass. Here, incompletely reacted sand raw materials can be seen in top left and centre, where (a) mineral remains of alkali feldspar (soda-rich), plagioclase (soda-rich) and ilmenite can be seen, (b) a fused boundary between partially reacted minerals and glass matrix can be observed, (c) a few voids retain the shape of dissolved minerals, and (d) the surrounding small bubbles which have not been incorporated into the fused glass due to the higher viscosity of this incompletely melted area. It can also be seen in Fig 2 that there are colourants (lead tin oxide in this case) or newly formed crystals (e.g. sodalite). These un-melted mineral remains, voids or bubbles, newly formed crystals and in some cases the colourant crystals, explain the opaque nature of m-Na-Al glass, which scatter the light and do not transmit it through the glass.

**Fig 3:**
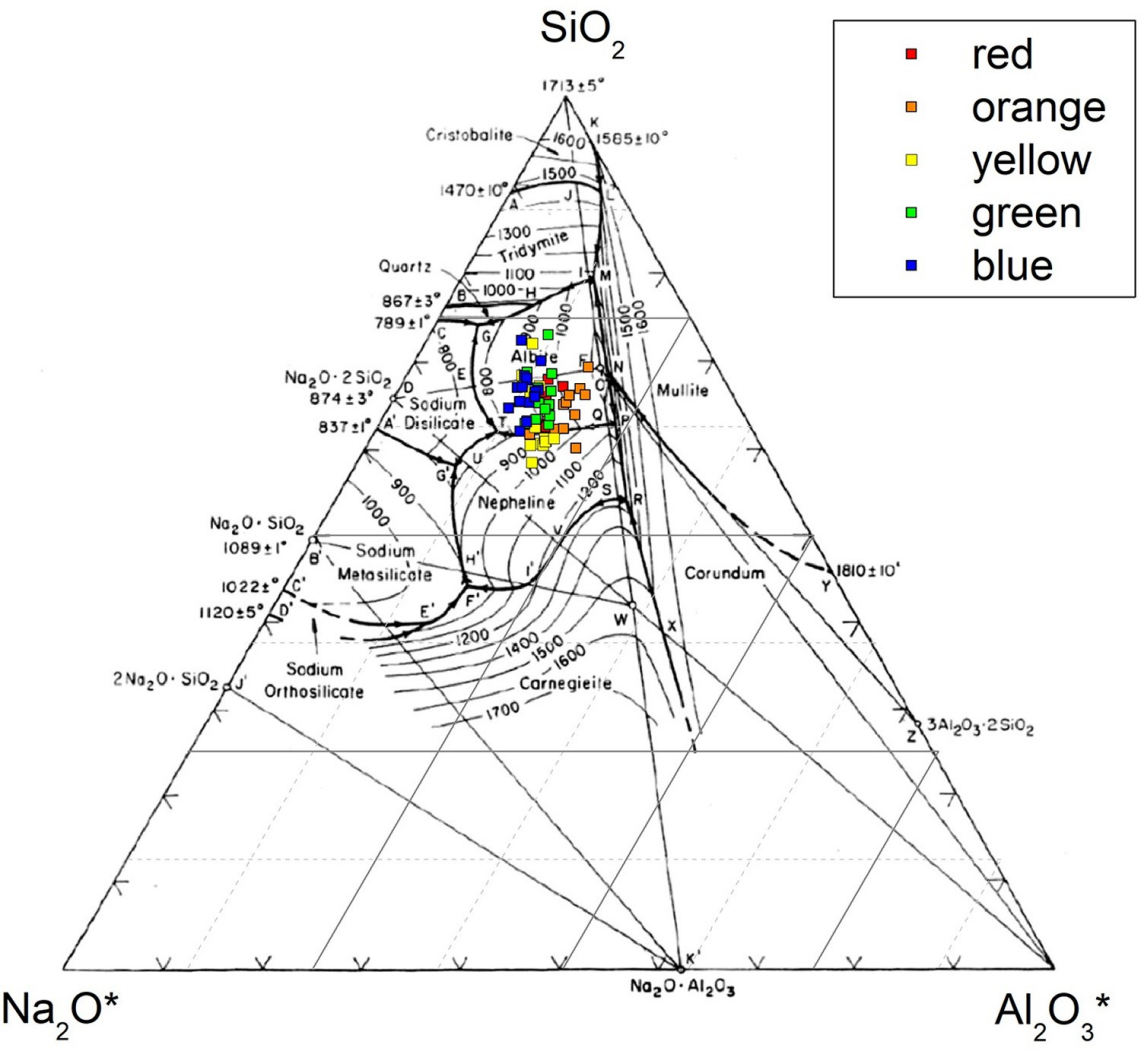
A plot of m-Na-Al glasses in the Na_2_O-Al_2_O_3_-SiO_2_ phase diagram. The oxides from the base composition are further reduced into three components (SiO_2_, Al_2_O_3_* and Na_2_O*)–the FeO, MgO and CaO are transmuted to Al_2_O_3_ (labelled Al_2_O_3_*), using a transmuting factor of 0.71, 1.26 and 0.91, respectively (Rehren 2016, pers. comm.). The K_2_O is incorporated into Na_2_O (labelled Na_2_O*) transmuted by the factor of 0.66. The base phase diagram is from Levin and McMurdie [23].

The use of a feldspar-rich sand suggests that the melting range of this type of glass may be low. This could be demonstrated by plotting these samples into the Na_2_O-Al_2_O_3_-SiO_2_ *quasi-*ternary phase diagram, as shown in Fig 3. Although these diagrams present a very rough approximation of melting temperatures as archaeological glasses are much more complex than a simple three phase diagram, they do provide a general indication of relative melting temperatures if not absolute ones and so should be interpreted with caution. Generally the samples are dispersed in the albite region of the diagram, roughly close to the isothermal section of 900–1000°C. An operational temperature below 1000°C for manufacturing these glass samples is likely. This can probably be further supported by the relics of incompletely melted feldspar as well as the ethnographic observation of glassmaking in northern India, where a production temperature of around 900°C is observed for making high alumina glass [15].

Apart from the glass matrix, the microstructure also reveals abundant information related to the use of colourants and colouring process. These are presented below.

### 4.2. Red glass

The red glass samples are coloured by cuprite (Cu_2_O) but are reported as CuO here. A CuO content between 0.8–1.6 wt% is found in the samples. In comparison to other colours (except for orange), slightly elevated level of FeO (1–2.5 wt%) are found in the red samples. The glass matrix exhibits a homogeneous glass composition, but shows a heterogeneous distribution of bubbles, zircon, aluminosilicate minerals and, more importantly, copper-containing inclusions.

**Fig 4:**
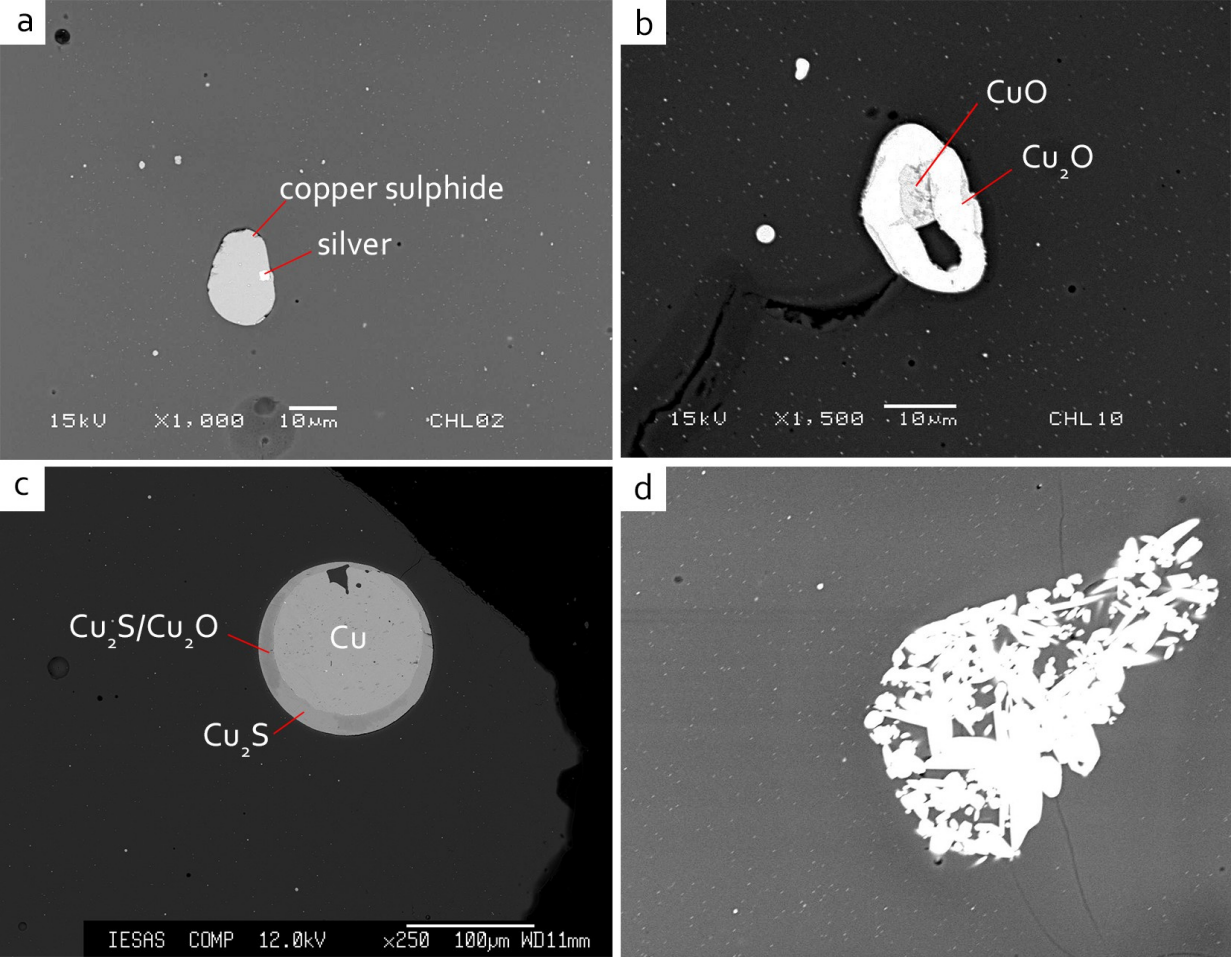
BSE image showing the relics in red glass, in which (a) silver particles are observed in copper sulphide inclusion (JXL02), (b) a copper oxide inclusion showing partial reduction process (JXL10), (c) a metallic copper inclusion showing partial oxidation (GS004), and (d) a cluster of needle-like tin oxide remain the crude cassiterite shape (JXL22).

SEM-EDS analysis reveals that these copper-containing inclusions are either copper sulphide or copper oxide. The former is more frequently found than the latter, this is because that copper oxide is more easily dissolved into the glass melt. The inclusions are irregularly shaped, with a size of about 10–30 μm, and often surrounded by bubbles or voids. The irregular morphology and the different sizes of these inclusions might suggest incompletely decomposed relics, rather than the recrystallisation of copper-containing crystals. A tiny silver particle is found embedded in a copper sulphide crystal in JXL02 (Fig 4A). Whilst in JXL10, a copper relic shows a partial reduction process, in which the inner cupric copper oxide (oxidised, with Cu ~38 at% and O ~42 at%) was encompassed by an outer cuprous copper oxide (reduced, with Cu ~50 at% and O ~27 at%) (Fig 4B). In sample GS004, a large copper inclusion of around 100 μm is observed (Fig 4C). EPMA analysis shows that this is a metallic copper surrounded by a rim of copper sulphide and partially oxidised copper sulphide/copper oxide. Slight decomposition can also be seen in the inner part of the prill. This inclusion is unlikely to be a newly formed crystal due to its particularly large size, and thus this may be the relic of colourant raw material. It is therefore probable that the red colourant raw materials here contain both oxidic and sulphidic copper.

The use of copper-tin alloy seems to be less common in the red samples. Most samples have a SnO_2_ content below 0.08 wt%, although slightly greater contents of around 0.15 wt% are detected in three samples JXL10, JXL35 and GS074. Acicular or nodular tin oxide are found in a few samples, and in some cases the partially decomposed tin oxide retains the shape of a cassiterite mineral (Fig 4D). These results suggest that copper-tin alloy may not be the main ingredient used for producing red glass here, and the tin contents may be accessory impurities introduced with copper.

### 4.3. Orange glass

**Fig 5:**
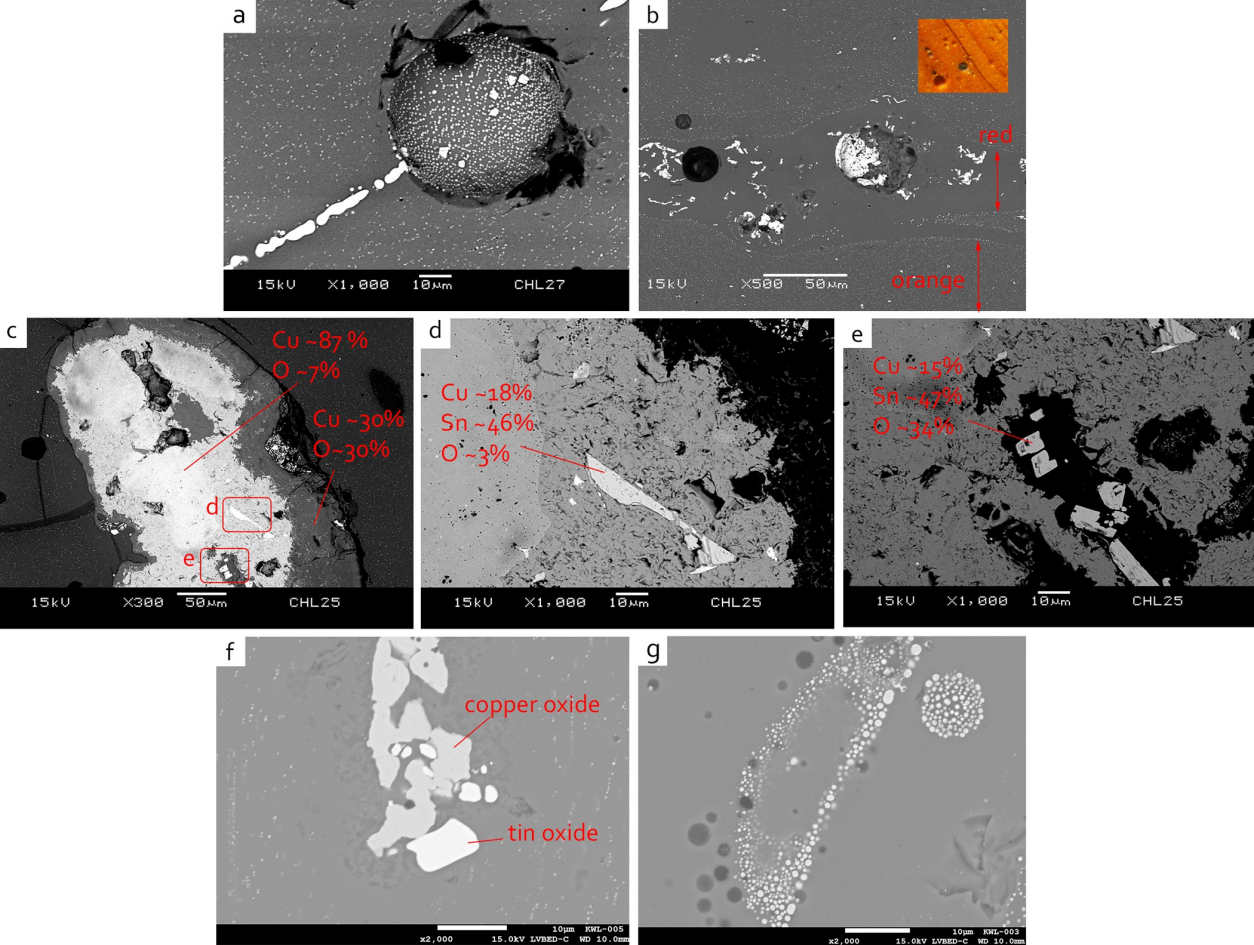
BSE image showing the microstructure of orange glass, in which (a) copper-bearing crystals of micro size distribute linearly over the glass matrix or fill within round voids (JXL27), (b) large copper oxide crystals are found in the red striae, while the particles of sub-micron size spread over the orange matrix (JXL24), (c)-(e) oxidised bronze prill was observed (JXL25, wt%), (f) a cluster of copper oxide and tin oxide suggesting decomposition from bronze (KWL005), and (g) large metallic copper crystals are confirmed by EDS (KWL003).

The orange glass is coloured by cuprite or metallic copper. In comparison to the red glass, a greater amount of CuO is found in the orange samples, from 3.1–7.8 wt%. The glass matrix is dispersed by relatively fine copper-containing particles of sub-micron size, probably metallic copper, but too small to be confirmed by SEM-EDS. Linear striae of larger copper-bearing crystals, a few micrometres in size and parallel to the perforation hole, are often observed (Fig 5A), which may result from the pulling motion in drawn beadmaking manufacture. Round voids filled with copper-containing crystals frequently occur along or near the striae, and small bubbles are often distributed near the crystals (Fig 5A). SEM-EDS analysis reveals these copper-containing crystals are copper oxide or metallic copper. Greater amounts of FeO (3–8 wt%) are found in the orange samples, suggesting the possibility of intentional introduction to increase the reduction of metallic copper (see 4.1).

The colouring mechanisms of orange glass and red glass are attributed to cuprite or metallic copper [24]. Here, the reddish striae sometimes exhibit within orange glass, and their varied microstructure is revealed by SEM-EDS. Fig 5B shows sub-micron copper particles distributed across the orange glass matrix. In the red part (darker grey), larger *copper oxide* crystals can be clearly observed, either distributed as dendritic forms or as clusters of partially decomposed inclusions within the voids. The CuO concentration is also greater in the orange matrix (~11 wt%) than in the red striae (~9 wt%). This indicates that the colouring mechanism of red and orange glass could be attributed to the grain size, the CuO concentration, and also the degree of reduction of the copper oxide. It is also worthy of pointing out that the dendritic forms of cuprite in the red striae here are not seen in the other monochrome red glass beads reported above. This may be a result of the greater copper content in the orange glass, which promotes the nucleation of cuprite crystals [25].

The analytical results indicate at least two different types of copper-colouring raw materials may have been used for producing the orange glass–tin-containing copper and tin-free copper. All the three samples from Jiuxianglan and three samples from Kiwulan (KWL001-o, KWL001-o and KWL005) have greater SnO_2_ contents between 0.4–1.2 wt%, while negligible amounts of SnO_2_ are found in the other two Kiwulan samples (KWL003, KWL004). Further microstructural analysis suggests tin-containing copper may be used in samples with greater SnO_2_ contents.

A striking find of a large inclusion containing Cu and Sn, around 450 μm in diameter, is found in JXL25 (Fig 5C–5E). Chemical analysis by EDS reveals that the inner part contains a relatively high Cu content (~87 wt%), while the outer shell is oxidised, with Cu contents lower than 30 wt% and elevated levels of Si and O. Within the inclusion, the rectangular or square crystals of copper-tin show enrichment of tin (~47 wt%) than copper (~15 wt%), likely in oxidised form (with oxygen in 3–34 wt%). These results indicate that the inclusion may be relate to bronze (Cu-Sn alloy), probably oxidised/solid state burning of bronze scraps or by-products or waste associated with bronze production. Evidence of this is also seen in KWL005. A cluster of copper oxide and tin oxide crystals suggests this may be decomposed bronze as well (Fig 5F).

In comparison, the two Kiwulan samples (KWL003, KWL004)) with negligible SnO_2_ contents have a different microstructure. Fine copper-containing sub-micron particles are widely distributed over the glass matrix, with larger sized copper-based crystals distributed linearly in the two samples. SEM-EDS analysis confirms that these large crystals are metallic copper, with Cu contents close to 100 wt% (Fig 5G). No tin oxide crystals or Cu-Sn clusters are found in both KWL003 and KWL004. This suggests a tin-free copper may have been used in these orange samples.

Different copper sources can potentially also be observed from the minor and trace elements here, in particular PbO, Ni, Zn, As and Sb (Table 2). Smaller amounts of PbO (<0.02 wt%), Co (<5 ppm), Ni (<30 ppm), Zn (<30 ppm)), As (<22ppm), Sb (<7ppm) are seen in KWL003 and KWL004 (tin-free copper samples). In samples with tin-containing copper from both Jiuxianglan and Kiwulan, there are often greater amounts of PbO, Zn, As and Sb. However, there are variable contents of Co, Ni and Zn between Jiuxianglan and Kiwulan samples, and even *within* Kiwulan samples. The Jiuxianglan samples (JXL24, JXL25, JXL27) generally have lower Co (11–22 ppm) and Ni (14–71 ppm), but relatively high Zn (>735 ppm) compared to the tin-rich Kiwulan samples (KWL001-o, KWL002-o, KWL005). Within the three tin-rich samples from Kiwulan, two samples (KWL001-o and KWL002-o) have particularly high Co (> 55ppm) and Ni (>140ppm) and moderate Zn. As for KWL005, the concentration of Co and Ni is similar to Jiuxianglan samples, but Zn is low (75ppm).

**Table 2:** Chemical composition in relation to the use of copper colorant.

Sample	CuO (wt%)	FeO (wt%)	SnO_2_ (wt%)	PbO (wt%)	Co (ppm)	Ni (ppm)	Zn (ppm)	As (ppm)	Ag (ppm)	Sb (ppm)
JXL24	7.45	2.55	1.24	1.55	21.69	14	1774	297	48	50
JXL25	4.75	1.94	0.47	1.43	10.65	57	1103	93	45	92
JXL27	5.20	1.70	0.39	0.90	10.89	71	735	178	37	120
KWL001-o	6.01	2.86	1.34	1.88	55.47	277	554	179	21	65
KWL002-o	7.81	2.84	0.35	1.96	156.83	141	787	616	69	36
KWL003	3.13	1.22	0.02	0.02	5.01	32	30	22	20	7
KWL004	4.07	1.59	0.01	0.04	5.46	25	19	12	7	3
KWL005	4.95	1.61	0.71	0.69	15.69	77	75	133	43	49

These results reinforce the suggestion that for the tin-rich samples different copper sources may have used in beads found at Jiuxianglan and Kiwulan. Within Kiwulan, it is also noteworthy that the bead style of KWL001-o and KWL002-o are not the same as KWL005. KWL001-o and KWL002-o are double-layered glass beads covered with an orange glass surface, while KWL005 is a fully orange bead. Thus the varied minor and trace elemental pattern suggests that, despite all being coloured by tin-containing copper, there *may* be different production areas and hence supply chains for the two styles of glass beads at Kiwulan.

### 4.4. Blue glass

The blue glass is coloured by cupric copper, within the solution of glass network, rather than as the cuprite or metallic copper crystals as seen in the red and orange glass. Therefore, in contrast to the red and orange glass, relics or crystals associated with colourant are less common in the microstructure.

The blue glass has a CuO content of 0.5–2 wt%. The matrix of blue glass is homogeneous, often with dispersed bubbles and mineral remains such as zircon or feldspar. The use of tin-containing copper could be suggested by the cluster of copper oxide and tin oxide in JXL28, and by acicular tin oxide crystals in a few blue samples. The SnO_2_, however, is below 0.15 wt%, suggesting the low solubility of tin oxide in glass melt.

**Fig 6:**
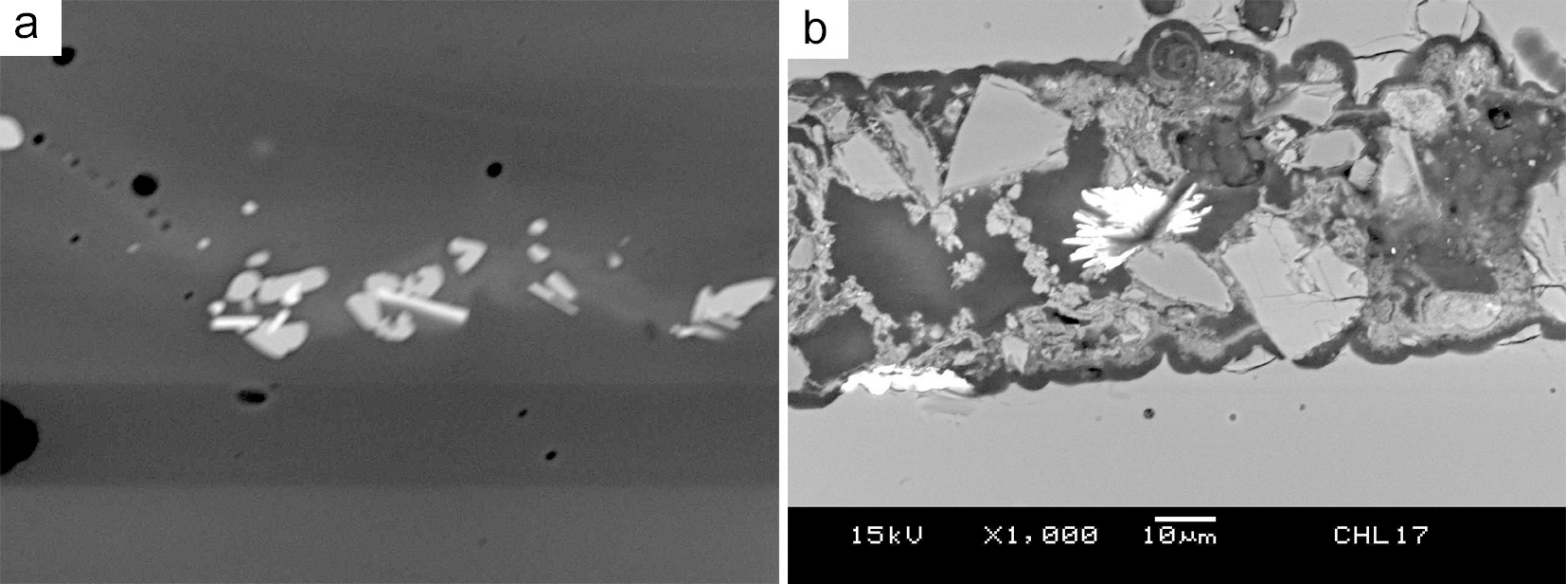
BSE image the microstructure of blue glass, in which (a) clusters of copper oxide (grey) and tin oxide (bright) (JXL28) and (b) the prismatic barite inclusions (JXL17) are observed.

Prismatic barite (BaSO_4_) inclusions are found in JXL17 (Fig 6). As yet the raw materials used to colour these beads are unclear, although an association with sulphur-based copper is likely, and thus this inclusion may be a relic from less purified copper-containing raw materials.

### 4.5. Yellow glass

The yellow glass is coloured by lead tin oxide as colourant crystals distributed over the glass matrix, which also contributes to the opacity. SEM-EDS analysis indicates there are always a few wt% of Si within the lead tin oxide. Thus the actual chemical formula is Pb(Sn,Si)O_3_ rather than PbSnO_3_. The yellow glass shows an uneven distribution of PbO content in the glass matrix. The crystals are dispersed throughout the matrix, particularly those related to colourant. The PbO composition in the matrix varies from 1–8 wt%, and SnO_2_ is below 1 wt%, which suggests the SnO_2_ is relatively insoluble in the glass melt. Slightly greater mean concentration of BaO is found in the Jiuxianglan samples, and this could be interpreted as impurities introduced by the Pb-containing materials [14].

**Fig 7:**
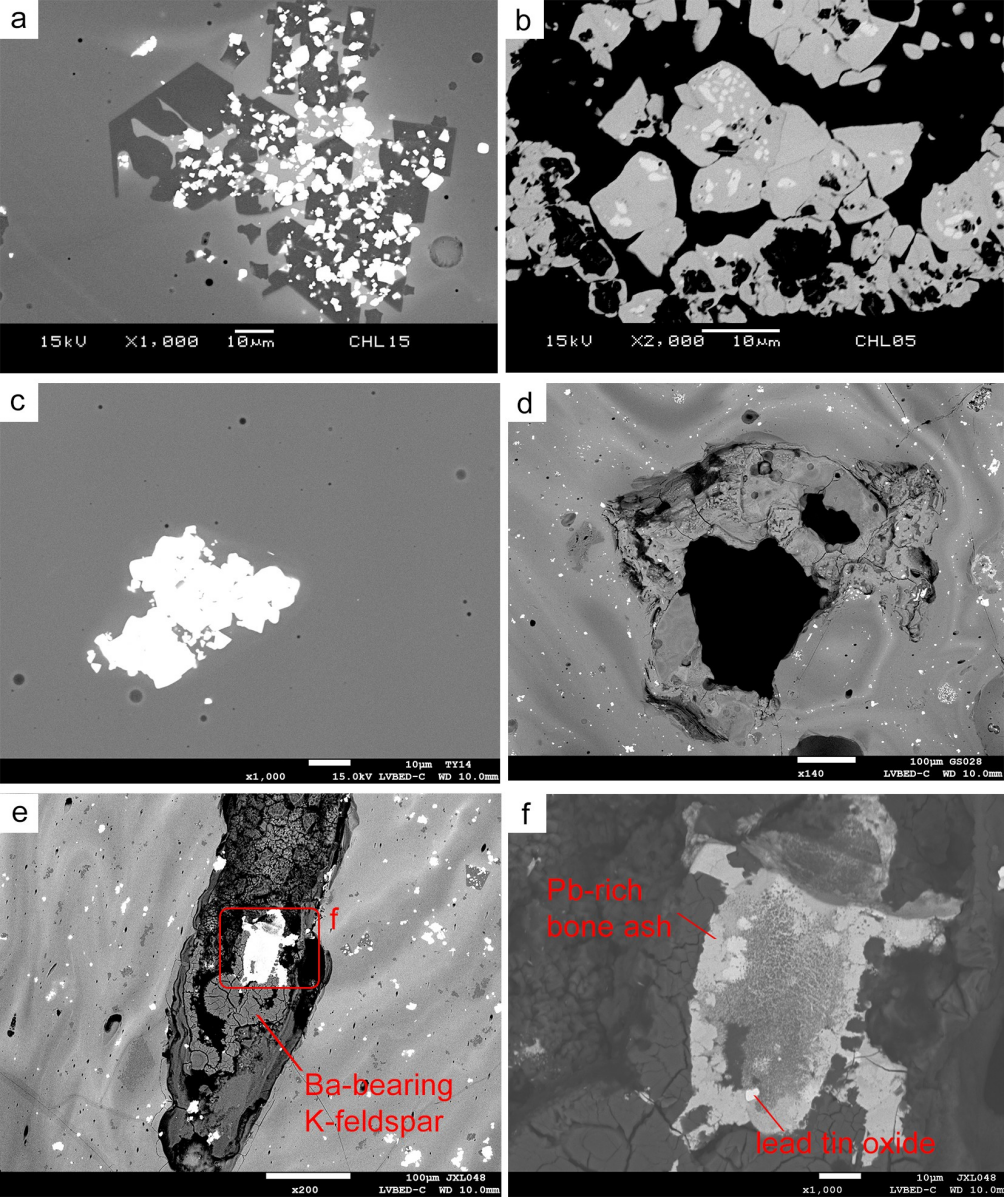
BSE image showing microstructure of yellow glass, in which (a) clusters of tiny lead tin oxide crystals are frequently found with large and newly formed nephelines in Case 1 (JXL15), (b) the cores of Pb(Sn,Si)O_3_ exhibits possible Pb_2_SnO_4_ remnants (JXL05), (c) lead tin oxide clusters solely, without any nepheline, in Case 2 (DY14), (d) porous bone ash is observed (GS028), and (e)-(f) Ba-bearing K-feldspar and lead tin oxide is found in the bone ash-containing samples, and the bone ash is particularly high in PbO (>60wt%) (JXL48).

Generally, two different microstructures are noted. In Case 1, lead tin oxide commonly clusters with nepheline (NaAlSiO_4_), scattering in the bright matrix with an increased amount of PbO (Fig 7A). Sometimes, hexagonal sodalite (Na_8_Al_6_Si_6_O_24_Cl_2_) is found near the cluster, and sometimes within the lead tin oxide agglomeration. Within the cluster, the size of lead tin oxide is usually a few micrometres, while nepheline is ten more times larger. The regular tetragonal and hexagonal shapes of nepheline, together with its clear grain boundary between the matrix, indicates that this is a newly formed crystal rather than an un-melted relic. In the core of the lead tin oxide crystal, occasionally there are areas showing elevated level of Pb but decreased Si, possibly remnants of Pb_2_SnO_4_ (Fig 7B). In the Pb-poor dark matrix, sodalite crystals can also be found near the sodium aluminosilicate relic which is not fully melted. In Case 2, the distribution of PbO is more homogenous than in Case 1. The agglomeration of lead tin oxide crystals is also scattered in the glass matrix, but no newly formed nepheline or sodalite crystals were found as a cluster (Fig 7C). The two types of microstructures may suggest different colouring processes or heat treatments (see discussion).

In 2 yellow glasses from Guishan (GS028 and GS029) and 1 yellow glass waste from Jiuxianglan (JXL47), bone ash may have been added with lead-tin yellow colourant along with aluminosilicate materials (see Wang et al. [22]). The bone ash shows a porous texture (Fig 7D), containing variable contents of CaO 10–31 wt% and P_2_O_5_ 21–33 wt% and some SiO_2_ and Al_2_O_3_. Ba-bearing K-feldspar (BaO ~ 3 wt% and Ba ~0.5 at%) is occasionally found in these yellow glasses containing bone ash, but not in other yellow samples studied here. The BaO and K_2_O contents in the glass matrix, however, fall in the same level as other yellow samples not containing bone ash.

Small crystals of lead tin oxide are often surrounding or within the bone ash lump and some are particularly rich in PbO (Fig 7E and 7F). The purpose of using bone ash in the yellow glass remains unclear, although increasing the opacity could be one potential reason.

Most lead tin oxide crystals have an equant shape. However, in GS028, the presence of acicular lead tin oxide may suggest incomplete decomposition of the lead-tin calx to cubic lead tin oxide [22:8]. Lead tin oxide is also clustered with sodium aluminosilicate in these bone ash-containing samples. Here, sodalite is more commonly found than nepheline. Taken together the presence of Ba-bearing K-felspar, it seems reasonable to suggest the raw materials used for producing the bone ash-containing samples may be different from other yellow glasses, with the former having more Cl-based ingredients and probably containing varied feldspar components.

### 4.6. Green glass

The colouring of green glass is a mixing effect of blue and yellow glass, that is, the blue hue by ionic cupric copper and the yellow tone by lead tin oxide crystal. Occasionally yellow striae can be observed by optical microscopy on the bead surface. Chemical analysis shows the samples contain CuO of 0.5–1.5 wt%, PbO of 1–6 wt% and SnO_2_ below 0.5 wt%.

**Fig 8:**
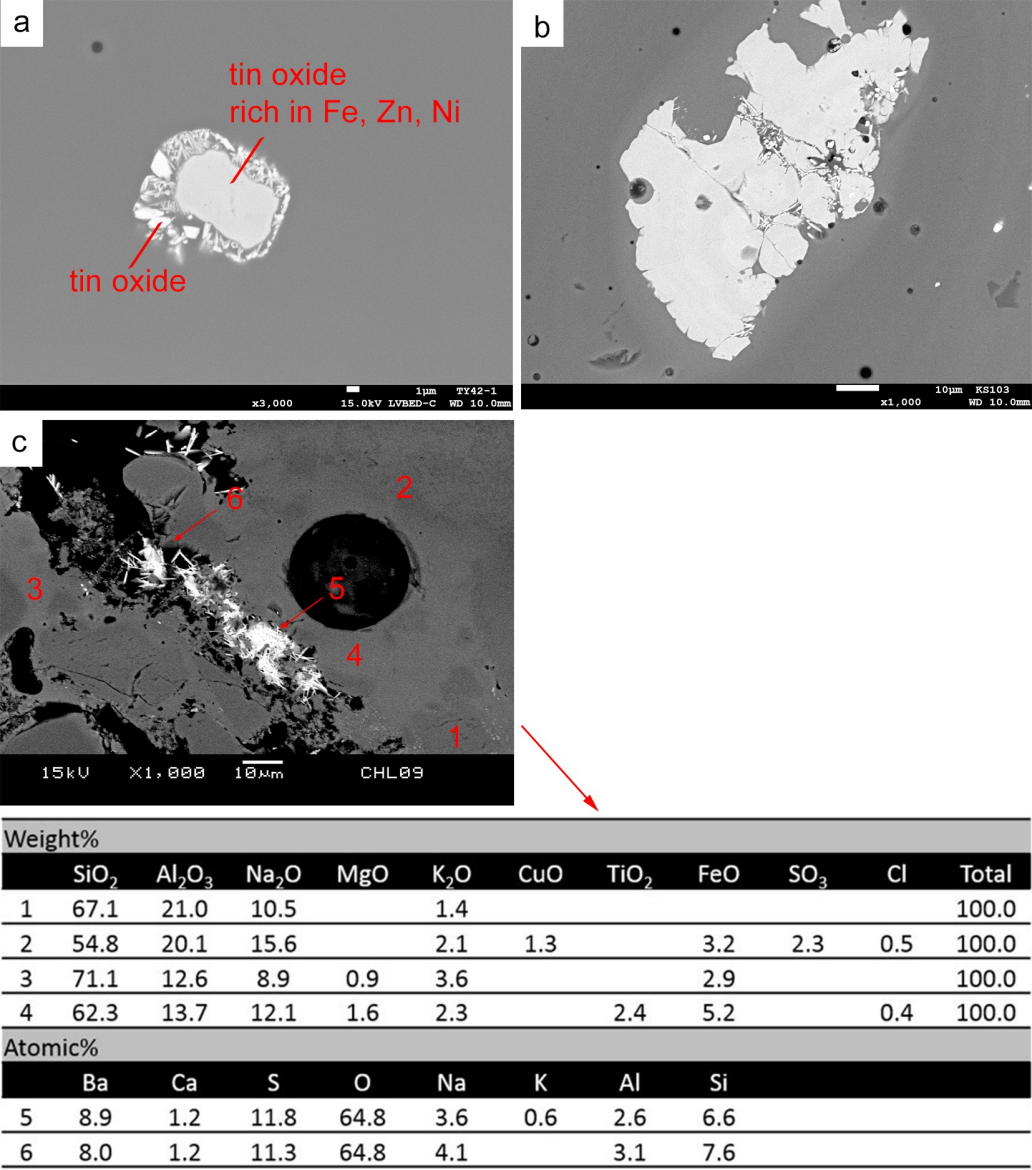
BSE image showing microstructure of green glass, in which (a) a tin oxide rich in Fe, Zn and Ni is found (DY42-1), (b) a tin-rich inclusion containing Ca, Al and Si is observed (GS103), and (c) a visible red inclusion showing inhomogeneous distribution of CuO, TiO_2_, FeO and SO_3_, as well as prismatic barite crystals and partially melted sodium aluminosilicate (JXL09).

Similar to the yellow glass, the microstructure of green glass shows an uneven distribution of PbO content in the matrix, and small crystals of cubic lead tin oxide scatter within the glass matrix. In some cases, lead tin oxide also clusters with nepheline or sodalite, while in others lead tin oxide agglomerates as a single cluster, without any nepheline or sodalite. Other mineral relics related to the colouring raw materials are observed. This includes copper oxide and tin-rich inclusions showing marks of decomposition. These tin-rich inclusions contain variable numbers of impurities, suggesting that these are not a re-crystallised product but un-melted relics. For example, Fig 8A is a tin oxide inclusion rich in Fe, Zn, Ni and Sn, and Fig 8B is a tin-rich inclusion containing Ca, Al and Si. The tin-rich inclusions may be accidentally introduced with the lead-tin calx or copper colourants during the colouring process.

JXL09 contains a visible red inclusion. SEM-EDS analysis reveals that this micro-area exhibits a different microstructure from the green glass matrix. There is no PbO within the matrix and no clusters of lead tin oxide (Fig 8C). In contrast, the glassy matrix shows an inhomogeneous distribution of CuO, TiO_2_, FeO and SO_3_. Within the inclusion, there are prismatic crystals of barite and several relics of partially dissolved sodium aluminosilicate. It is tentatively thought that this inclusion may be related to the use of metallurgical by-products or waste, and thus suggests the cross-craft interaction between glass production and metallurgy (see discussion).

## 5. Discussion: Raw materials, production technology and cross-craft interaction

This analysis has contributed to a greater understanding of the raw materials used to produce and colour type 1 m-Na-Al glass, currently thought to originate from South Asia. The common presence of sodium-rich alkali feldspar, plagioclase and other accessory minerals such as zircon and ilmenite, supports the assumption made by Dussubieux, Gratuze and Blet-Lemarquand [2] that granite sand may be used for primary production, containing more pure quartz than sodium-rich feldspar. The fact that some feldspar relics are not completely melted further demonstrates that the operational temperature may be low, not exceeding 1000°C, and the frequent mineral remains indicate the sand used is impure and perhaps not carefully refined. As the sand contains principally quartz and has impurities other than sodium-rich feldspar, which is found in insufficient quantities in the sand to provide an adequate flux, additional soda flux is necessary to facilitate the melting. More importantly, these results have provided abundant new data, in conjunction with an understanding of different mineralogies, geologies and raw materials, for the use of a variety of different colouring agents which are often used in novel ways and may indicate different provenances for beads of different colours found in Taiwan.

### 5.1. The colouration of red, orange and blue glass

Many of the different coloured glasses used copper as a base for colour production. This includes red, orange and blue and to some extent green glasses. For copper-based colourants, this study reveals the use of a variety of ingredients as raw materials, notably two broad categories as tin-containing and tin-free copper, and also the skilled control of the redox conditions, to produce the red, orange and blue glass colours.

#### 5.1.1. Tin-containing copper compounds

Tin-containing copper compounds were added to produce orange glass, made in reducing conditions, and in blue glass in oxidising conditions. The primary raw materials may be bronze in metallic form. However, metallic bronze is insoluble in glass melt, and thus the metal/alloy is very likely to be heated and/or oxidised (as seen in JXL25, Fig 5C–5E) in order to produce copper oxide which can be dissolved into the glass. Its use was rarely seen in red glass in this study.

Tin is not an essential component for producing *blue* glass. Thus its presence in blue glass, particularly as a cluster with copper oxide, suggests unintentional introduction with copper. This indicates that the colouring agent of blue glass may be ‘burnt’ and/or oxidised bronze scraps (copper-tin alloy) or by-product of bronze metallurgy.

In the case of tin-bearing *orange* glass (JXL24, JXL25, JXL27, KWL001-o, KWL002-o, KWL005), a bronze source is also likely. However, here the intentional selection of tin-containing compounds needs consideration, as tin can behave as the reducing agent for the generation of cuprite or metallic copper crystals in the orange glass, or to induce their nucleation and precipitation [26–29]. The elevated PbO contents in the tin-containing orange glass therefore may be accidentally introduced with tin bronze components. It has been suggested that a high PbO concentration can also promote the crystallisation of cuprite in the glass [25,30]. In this research the lower amount of PbO (< 2 wt%) indicates the effect of PbO in cuprite precipitation may be minor. In contrast, the greater FeO contents in the orange glass, compared to the blue glass, suggests an additional source of FeO may have been selected to achieve a more reducing condition for the precipitation of cuprite or metallic copper [30]. However, although the effect would be apparent, the ancient glassmaker may not have been aware of the role of tin, and possibly lead, in the bronze for producing the orange glass, but was conscious that the combination of specific colouring additives produced a specific colour. Therefore, oxidised bronze scraps and an unknown iron-containing colouring raw material may have been deliberately chosen and processed for making orange glass but not other colours.

#### 5.1.2. Tin-free copper and the self-reduction model

In terms of the use of tin-free copper, a mixture of oxidic copper and sulphidic copper compound is likely to be introduced to most red glass samples. Oxidic copper, such as scaled or hot-oxidised copper, may be the major colourant component in this case. The high proportion of copper sulphide droplets seen in some samples here is because most copper oxide would be dissolved into the glass before forming copper oxide crystals, thus the presence of copper sulphide, in crystalline forms, is exaggerated as it is more apparent. The partially reduced copper oxide relic shown in Fig 4B, with CuO in the core and Cu_2_O in the outer surface, further indicates that the materials initially added are more oxidised. It is tentatively suggested here that the addition of metallurgical by-products such as matte, which is principally composed of copper and sulphur, may be one possibility for the presence of sulphidic copper. Matte is an intermediate product of smelting sulphidic copper or copper-iron ore. Sometimes iron occurs in matte as part of the solid solution within the matte prill (see for example Bennett [31]; Cadet [32]). The iron content may thus be introduced into the glass melt, resulting in slightly elevated levels of FeO in the glass. However, other sources of sulphidic copper may also be possible. Further investigation and experimentation using matte as colouring raw materials, and any related preparation process, is required.

Alternatively, using a sulphidic/oxidic ore source as colouring raw materials is possible but considered less realistic here. The occurrence of crude copper oxide inclusions might suggest an oxidic copper ore, such as malachite or azurite, and the copper sulphide might be related to sulphidic ore, such as covellite, chalcocite or chalcopyrite. However, most copper ores are relatively ‘dirty’, containing too many ‘impurities’, and thus it would be time consuming and inefficient for the craftspeople to remove it. For example, accessible copper deposits in Southeast Asia often co-occurred with iron, gold or tin in various ore forms [33]. Direct introduction of a sulphidic/oxidic ore would inevitably bring ‘impurities’ into the glass melt; these are absent in the glass samples here. Thus, the use of raw copper ores is regarded as less practical and unlikely in these samples.

Copper smelting produces unwanted slag, which could be another potential raw material. However, the introduction of slag is also considered less likely, as it will introduce crystalline phases such as fayalite or magnetite into the glass melt (see for example Peake and Freestone [34]). The absence of these types of inclusions in the glass here indicates that slag, waste of copper smelting, was not added.

The co-occurrence of oxidic copper and sulphidic copper raises further discussion in terms of the colouration of the red glass. A mechanism similar to the co-smelting of oxidic and sulphidic copper for copper reduction in the red glass is tentatively proposed here. This could be tentatively supported by the partially oxidised copper sulphide/copper oxide droplet seen in GS004 (Fig 4), which is similar to the desulphrised copper droplet of matte prills observed by Hauptmann, Rehren, and Schmitt-Strecker [35]. The co-smelting of copper sulphide and copper oxide, which requires a continuous air supply, would lead to the self-reduction of cuprite and metallic copper in the red glass, although keeping a constant forced air supply may be challenging for ancient craftspeople to achieve. Furthermore, a good stoichiometric mixture of sulphidic/oxidic copper is required for the self-reduction process. This suggests that, firstly, the craftspeople had an excellent knowledge of the composition of the raw materials and processes when selecting/preparing proper raw materials and mixing the materials in the exact proportions. Secondly, the use of sulphidic/oxidic copper ore is most unlikely in this case, as it would be relatively difficult to obtain in the correct balanced mixture of sulphidic/oxidic ratios.

Similar colouring process, using mixed oxidic and sulphidic copper, might have been taken place in the tin-free orange glass samples (KWL003 and KWL004), with a higher degree of reduction of metallic copper compared to the red samples and, as mentioned above, with oxidic copper as the principal components. The greater level of FeO in the orange glass may also indicate that an additional FeO was introduced to increase reduction. Under a better control of the air flow and heat treatment, such as longer heating and cooling times, most copper sulphides would fully reduce to metallic copper, which explains the absence of copper sulphide relics and the presence of micro metallic copper crystals. Additionally, the reduction process generates gaseous SO_2_. An excess of SO_2_ gas in the glass melt produces small bubbles which can be captured by the viscous glass matrix. This may explain why more tiny bubbles are observed in these orange samples, in proximity or attached to the clusters or voids of the copper-containing inclusions/crystals. The self-reduction mechanism proposed here, when perfected, would allow red and orange glass to be produced in mass quantities for beads, and may explain the wide distribution of red and orange glass of m-Na-Al composition around the Indo Pacific region, when red glasses in particular are not common in other cultures.

### 5.2. The use of lead-tin yellow

Lead tin oxide is a common yellow pigment used in antiquity. Its production involves the calcination and mixing of lead- and tin-containing raw materials, with the addition of silica [36]. The yellow pigment can be introduced into raw glass as a colourant and opacifier [37]. Here, it is thought that the microstructure of Case 1 (lead tin oxide and nepheline clusters) and Case 2 (homogenously distributed lead tin oxide) may indicate different colouring process.

Case 1 may indicate that the lead- and tin-containing raw materials were introduced separately, or more probably as incompletely reacted lead-tin calx, into the raw glass (or low-fired glass frit). The glass sand used for producing m-Na-Al glass contains abundant sodium aluminosilicate (albite or plagioclase), and the SEM-EDS analysis in this research shows that a certain proportion of the sodium aluminosilicate particles are not fully melted. During the melting process, the Pb and Sn react with Si from the glass phase, or from the un-melted sodium aluminosilicate, to produce crystalline lead-tin yellow Pb(Sn,Si)O_3_. In the ternary phase diagram shown in Fig 3, the chemical composition of the glass matrix generally falls in the albite region. Excess PbO may have been added in Case 1, which dilutes the Si-contents in the local glass melt or the unmelted sodium aluminosilicate. The local chemical composition may have changed from an Si-rich phase (albite) towards an Si-poor phase (nepheline) (Fig 3), which facilitates the formation of nepheline, and results in an increasing amount of PbO in the glass matrix where the lead tin oxide and nepheline crystals agglomerate. This also explains the fact that most yellow glass samples fall towards the nepheline phase in Fig 3, as the matrix may be Si-poorer due to the release of excess PbO into the glass melt. Additionally, the glass raw materials may have contained chloride, sulphate or sulphide, which contains S and Cl. The formation of sodalite (Cl-containing sodium aluminosilicate, sometimes contains sulphur) in the dark matrix (Pb-poor) could be attributed to the reaction between sodium aluminosilicate (existing albite or newly-formed nepheline) and the sulphur- and chlorine-based ingredients [38,39].

In Case 2, the lead-tin yellow may be added as a single compound and finished product in which lead and tin raw materials were calcined, mixed and melted in advance [37]. It is also possible that the lead-tin yellow was introduced to a more vitrified glass frit which contains fewer relics of sodium aluminosilicate. As a result, the reaction between Pb, Sn, Si and sodium aluminosilicate minerals are hardly ever seen in Case 2.

In either case 1 or 2, crystals of tin oxide are absent. This may indicate that the colouring process of both glasses was carried out at a temperature below 1000°C, as lead tin oxide would probably be dissolved above 1000°C, leading to the recrystallisation of tin oxide which would produce white rather than yellow crystals, which was not observed here [40].

### 5.3. The barite and barium contents

A noteworthy find in this research is the occasional presence of barite in the blue and green glass. In particular, the large red inclusion containing barite crystals in the green sample JXL09 shows an enrichment of Cu, Fe and S, which may be associated with the use of sulphidic copper-iron raw materials. Copper oxide is used as colourant in both blue and green glass beads. As discussed in 5.1 as sulphidic copper may be used as colouring agent, the presence of barite in the blue and green glass thus may be a serendipitous occurrence with the sulphidic copper-containing raw materials, although tin-containing copper may have been used in some blue glass samples. The absence of copper sulphide relics in the blue and green glass may be attributed to the oxidation conditions; the copper sulphides are oxidised to oxidic copper and incorporated into the glass melt as ionic cupric copper. If this is the case, the presence of red inclusions containing barite, and varying levels of Cu, Fe and S, reinforces the assumption here that there may be a tradition of using sulphidic copper, in conjunction with oxidic copper, as colouring raw materials in the production of various hues of copper-coloured glass of an m-Na-Al composition.

The association of Ba with the Pb-containing colourant is also tentatively suggested in a few yellow glass samples from Jiuxianglan [17]. This association of Ba with the colourant raw materials further suggests that Ba contents in the m-Na-Al glass sub-type 1 need to be interpreted with caution, as Ba is regarded as one diagnostic element suggesting geological variations in sand and thus provenance [2]. Although a closer examination shows that there is no clear differentiation of the Ba contents in different colour groups in this research (except for the Jiuxianglan samples), the microstructural results demonstrate that a small amount of Ba may be attributed to the impurities of colouring raw materials rather than fully acquired from the sand. Whether or not this indicates the use of varied sources/types of colouring agents in different sub-types of m-Na-Al glass which also could be linked to provenance, however, is a question for future investigation and discussion.

### 5.4. Future perspectives on the reconstruction of m-Na-Al glass production and cross-craft interaction with copper metallurgy

Most of our current understanding and the focus of many research projects is the elucidation of primary production and provenance of m-Na-Al glass in South Asia. The microstructural data discussed here echoes previous discussions made by Dussubieux and her colleagues based on the chemistry of glass and the elucidation of the raw materials for primary production. The heterogeneity of microstructure seen in this research not only supports the assumption of using less refined sand, but further indicates that the pre-treatment of raw materials may be less standardised, and the melting condition less controlled to obtain a high-quality glass.

More significantly, this research has shown evidence for the colouring process which is seldom reported and discussed in current archaeological and ethnographic studies. At present, it is unclear whether colouring took place in the same workshop where the raw glass was made. The ethnographic records in South Asia mostly suggest that raw glass was purchased from other workshops for melting, or waste glass was recycled for glassworking, without further indication of colouring (e.g. Sode and Koch [15] and Kanungo [20]). However, in the documentary ‘Glassmakers of Herat’, shot in Afghanistan in 1968, it clearly recorded that the glassmakers heated copper scraps to produce oxidised copper scales and then added these to the raw plant-ash glass melt as colourant in a powdered form [41: 00:14:15].

This research shows the complexity of the selection and manipulation of colouring raw materials in the m-Na-Al glass. The study of glass microstructures alongside the chemistry of glass can therefore be used in conjunction to identify and separate technologies in order to trace patterns of circulation and exchange of shared knowledge of glass colouring in different regional production centres around the Indo-Pacific region, and illustrates the potential of this methodology to explore specialisation in glass colouring workshops.

Additionally, this research has demonstrated specific cross-craft interactions between glass and copper workshops in the Indo-Pacific region, although as yet it is not possible to determine the provenance of this glass and hence the location of the workshops. The use of metallurgical by-products or alloy scraps, potentially added as oxidised scales, in glass colouring has been suggested in glasses from a variety of contexts and periods from Late Bronze Age Egyptian, Roman, Byzantine and Anglo-Saxon periods and so this association of the two materials is not uncommon [34,42–45]. However, this is the first time it has been identified, through compositional and microstructural analysis in the m-Na-Al glass found around South China Sea, in the 1^st^ millennium AD. Despite the relatively low content of copper used to produce a variety of different colours in these glasses (CuO a few wt%), this research has shown craftspeople still needed access to the copper-containing raw materials through connections to copper production workshops and copper producers. In addition, they also appear to have had a sophisticated knowledge in the pre-treatment of raw materials, the preparation and addition of raw materials in the correct proportions, and the control of air supply to the furnace to produce the temperatures and redox conditions needed when using different types of copper-bearing colourants. The variety of copper-containing materials identified in the study sites discussed here, spanning a wide chronology, therefore demonstrates a long and continuous practice of cross-craft interaction, between glass and copper-based production in South Asia, or other areas where m-Na-Al glass production took place but which is yet to be identified archaeologically.

## 6. Conclusion

This research has presented new perspectives on the raw materials and production technology of m-Na-Al glass, specifically the sub-type 1 through chemical and microstructural analysis. A less refined sand, composed of mainly of pure silica but enriched in sodium-rich feldspar, was used for producing the raw glass, at a temperature below 1000°C. Although no glassmaking or glass colouring centres have been securely identified in prehistoric Taiwan in the archaeological record, glass beads of various colours from consumption contexts in Iron Age Taiwan provided a suitable assemblage for understanding their production. This research has shown that copper in various forms is by far the most widely used ingredient for red, orange, blue and green glass colouring. The analysis suggests the use of several copper-containing raw materials were used, such as ‘burnt’/heated oxidised bronze scraps or oxidised copper scales. Additionally, the presence of, for instance, lead tin yellow indicates the craftspeople are aware and had knowledge that a combination of different additives to the glass would produce specific hues or colours in glass. More significantly the glass microstructure suggests that a self-reduction process might have been applied for the red/orange glass production although this does require very careful control of a continuous and controlled air supply and sophisticated preparation/combination of a balance of raw material colourants. For the yellow colourant lead tin oxide may have been used using a variety of production paths, such as the addition of raw materials or through a further processing stage such as manufacture of a calx before addition to the glass.

The processing of these colouring agents requires not only the access to the raw materials, but the knowledge to identify and select suitable materials, understand how to prepare them to produce the desired colours and effects, the redox behaviour of different compounds under different temperatures, and the use of additives, in addition to the colourants, to enhance the colouring effect. As most colouring agents are metal compounds, the interaction or knowledge shared between glass and metal production therefore is likely around the Indo-Pacific region. Currently, the production of m-Na-Al glass sub-type 1 has only identified archaeologically in Sri Lanka, although the characterisation of this sub-type by chemical analysis and the wide distribution of this glass composition over time and space suggests there may be other regional production centres. The microstructural analysis provides complementary and important information for understanding the production behaviours of m-Na-Al glass. The relatively long chronology of the beads analysed here, and the variety of colouring raw materials identified, demonstrates an active and dynamic production network involving the exchange of information and materials through different glass and bead production stages. This research adds substantially to an understanding of the reconstruction of m-Na-Al glass production and circulation in the Indo-Pacific region, and shows a clear connection between pyrotechnological activities.

## Supporting information

S1 Table Full chemical composition of glass samples analysed in this research. (XLSX)
